# Structural Disorder within Paramyxoviral Nucleoproteins and Phosphoproteins in Their Free and Bound Forms: From Predictions to Experimental Assessment

**DOI:** 10.3390/ijms160715688

**Published:** 2015-07-10

**Authors:** Johnny Habchi, Sonia Longhi

**Affiliations:** 1 Aix-Marseille Université, Architecture et Fonction des Macromolécules Biologiques (AFMB), UMR 7257, 163, Avenue de Luminy, Case 932, 13288 Marseille, France; E-Mail: jh884@cam.ac.uk; 2 Centre National pour la Recherche Scientifique (CNRS), AFMB UMR 7257, 163, Avenue de Luminy, Case 932, 13288 Marseille, France

**Keywords:** paramyxoviruses, nucleoprotein, phosphoprotein, intrinsic disorder, induced folding, fuzzy complexes, protein-protein interactions, disorder prediction, molecular recognition elements, antiviral approaches

## Abstract

We herein review available computational and experimental data pointing to the abundance of structural disorder within the nucleoprotein (N) and phosphoprotein (P) from three paramyxoviruses, namely the measles (MeV), Nipah (NiV) and Hendra (HeV) viruses. We provide a detailed molecular description of the mechanisms governing the disorder-to-order transition that the intrinsically disordered C-terminal domain (N_TAIL_) of their N proteins undergoes upon binding to the C-terminal X domain (P_XD_) of the homologous P proteins. We also show that N_TAIL_–P_XD_ complexes are “fuzzy”, *i.e.*, they possess a significant residual disorder, and discuss the possible functional significance of this fuzziness. Finally, we emphasize the relevance of N–P interactions involving intrinsically disordered proteins as promising targets for new antiviral approaches, and end up summarizing the general functional advantages of disorder for viruses.

## 1. Overview of the Replicative Complex of Paramyxoviruses

Negative-stranded RNA viruses (NSRVs) are causative agents of a large number of human and animal diseases with some of them being identified as potential agents of bioterrorism, and several being included in the NIAID (National Institute of Allergy and Infectious Diseases) and CDC (Center for Disease Control and Prevention) priority pathogen lists. Research on these viruses, as well as efforts aimed at the development of vaccines and antiviral drugs, have been paid an increased attention for many years. NSRVs can be divided into viruses with segmented RNA genomes and those with non-segmented RNA genomes. The latter are grouped within the *Mononegavirales* order. Among the viral families belonging to this order is the *Paramyxoviridae* family. The Nipah (NiV), Hendra (HeV) and measles (MeV) viruses belong to the *Paramyxovirinae* sub-family within the *Paramyxoviridae* family, where the latter also embraces the *Pneumovirinae* subfamily. Based on distinguishing nucleotide sequence features, the MeV and the NiV and HeV and have been classified into two distinct genera, the *Morbillivirus* genus and the *Henipavirus* genus, respectively [1,2,3].

**Figure 1:**
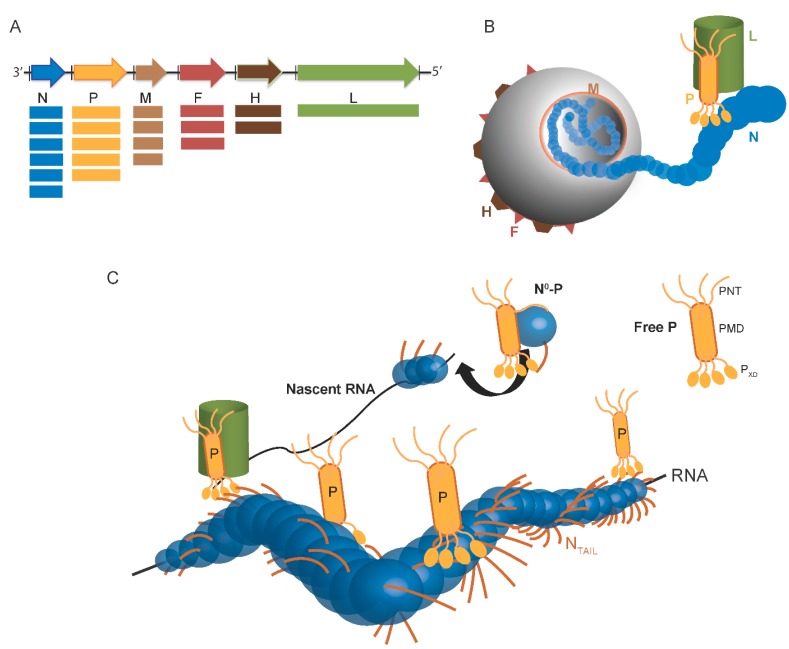
Scheme of *Paramyxovirinae* genome and viral particule. (**A**) Schematic representation of the genome of *Paramyxovirinae*. The negative-sense genomic RNA is presented in the 3′ to 5′ orientation. The open reading frames are represented by colored arrows and encode the nucleocapsid protein (N), phosphoprotein (P), matrix protein (M), fusion protein (F), attachment protein (H) and large polymerase protein (L). Vertical lines represent gene start and stop signals. Below the genome, shown is a schematic representation of the expression gradient of the encoded proteins as a result of the stop and re-initiation mechanism of the polymerase during transcription [2]; (**B**) Schematic illustration of the virion. The viral membrane is decorated by the F and H glycoproteins and M is located beneath the membrane. N is bound to genomic RNA and together with P and L forms the viral replication unit; (**C**) Schematic illustration of the *Paramyxoviridae* replicative complex. The RNA is represented as a solid black line. The neo-synthetized RNA is shown already partially encapsidated by N. The N and P intrinsically disordered regions are symbolized by lines. The extended conformation of the disordered regions is thought to allow the formation of a tripartite complex between N°, P and L required for nucleocapsid assembly. The P/L complex forms the RNA-dependent RNA polymerase (RdRp) complex that cartwheels onto the nucleocapsid complex via the X domain of P (P_XD_). P is shown as a tetramer to reflect the prevalence of this oligomeric state in paramyxoviral P proteins.

Paramyxoviruses display a pleomorphic structure. The viral particule contains the non-segmented, negative-stranded RNA genome, which encodes for at least six proteins (Figure 1A). The genome of paramyxoviruses indeed encodes the fusion (F) and the attachment (H) glycoproteins, which are responsible for virus entry, the matrix (M), which is required for virus assembly and budding, and the proteins of the replicative complex (Figure 1B). In paramyxoviruses, RNA transcription and replication require an intricate interplay between three components: the RNA-dependent RNA polymerase (L), the phosphoprotein (P), and the nucleoprotein (N). As in all *Mononegavirales* members, the genome is encapsidated by N within a helical nucleocapsid. The N:RNA complex, rather than naked RNA, is the template for both transcription and replication. During RNA synthesis, P tethers L onto the N–RNA template through the N-P interaction. The complex formed by the N, P and L proteins constitutes the viral replicative unit, and these proteins are necessary and sufficient to sustain replication of viral RNA in *Paramyxovirinae* (Figure 1C) [4,5].

The N protein is the most abundant viral protein (Figure 1A). Within infected cells, the N protein from *Paramyxoviridae* members is found in a soluble, monomeric form (referred to as N°) and in a nucleocapsid assembled form (referred to as N^NUC^) [6,7]. Following synthesis of the N protein, a chaperone is required to maintain this latter protein in the unassembled form in the cytoplasm. This role is played by the P protein, whose association prevents illegitimate self-assembly of N and retains N in the cytoplasm [8,9]. This soluble N°-P complex is used as the substrate for the encapsidation of the nascent genomic RNA chain during replication. The assembled form of N also forms complexes with P, either isolated (N^NUC^-P) or bound to L (N^NUC^-P-L), which are essential to RNA synthesis by the viral polymerase. Hence, the components of the viral replication machinery, namely P, N and L, engage in a complex macromolecular ballet (Figure 1C) (see [2,5,10,11,12,13,14] for reviews on transcription and replication).

Although *in vitro* L can synthesize short RNA transcripts using naked RNA as substrate in the absence of P [15], in infected cells P is required to stabilize L and to allow recognition of the N:RNA template. L is thought to carry out most (if not all) enzymatic activities required for transcription and replication, including nucleotide polymerization, mRNA capping and polyadenylation. It is found in low amounts in infected cells and is unstable unless bound to the P protein, thus making its full characterization challenging [16]. Since no functional paramyxoviral polymerase has been biochemically characterized so far, most of our present knowledge arises from bioinformatics studies. Among *Paramyxovirinae* members, the only exceptions are represented by the L/P complex from two *Paramyxovirinae* members, namely Rinderpest virus (RDV) whose polymerase has been partially purified [17], and Sendai virus (SeV) whose polymerase was shown to possess a methyltransefrase activity in its C-terminal region [18]. Among *Pneumovirinae*, the only exception is the L protein from respiratory syncytial virus (RSV) that could be partially purified and whose RNA polymerase activity was documented *in vitro* [19]. In addition, minireplicon studies allowed the identification within RSV L of a flexible hinge region tolerating insertion and demonstrated the crucial role of the GDNQ motif [20]*.*

Accordingly, most of our present knowledge of the replicative complex of paramyxoviruses concerns the N and P proteins. In the last decades, many efforts have been devoted to the molecular characterization of paramyxoviral N and P proteins. The N–P interaction has attracted much interest not only from a fundamental point of view, but also from a more applied perspective: in fact, since abolishing the N–P interaction prevents the recruitment of L onto the nucleocapsid template, this interaction is regarded as a potential target for antiviral approaches.

In the course of a thorough structural and functional characterization of paramyxoviral N and P proteins that made use of a wide range of bioinformatics and experimental approaches, we showed that these proteins are enriched in intrinsically disordered regions (IDRs) and that these IDRs play key roles in the formation of the tripartite N–P–L complex and in the establishment of a broad molecular partnership (for reviews see [11,21,22,23,24,25,26,27]). Our seminal observations on MeV P and N proteins fostered subsequent studies that brought awareness of the prevalence and conservation of structural disorder within paramyxoviruses N and P proteins thereby pointing to its functional significance.

Intrinsically disordered proteins (IDPs) and IDRs are widespread functional proteins/regions that lack stable structures under physiological conditions. Behind their inability to fold resides an important *raison d’être*, which is tightly coupled to specific features of their amino acid sequence. Indeed, a specific imbalance in the content of hydrophobic *vs.* polar residues in IDP/Rs, confers them the ability to populate a wide conformational space with conformations ranging from completely extended (*i.e.*, random coils, RC) to more compact (*i.e.*, pre-molten globules, PMGs, and molten globules, MGs). The nature of the conformational ensemble that IDP/Rs could sample is coupled to their function and, hence, to the interactions they establish with their partners. Indeed, in many cases, IDP/Rs fold upon binding leading to either stable complexes amenable to crystallization, or, more often, to fuzzy complexes [28], *i.e.*, complexes with significant residual disorder. These peculiar characteristics award IDP/Rs a number of advantages over folded proteins that promote their frequent involvement in particular functions, such as for instance, hubs in protein interaction networks and cell signaling (for a recent review on IDP/Rs see [29]).

In the current review, we provide a detailed description of the molecular information that exist to date on the N and P proteins from paramyxoviruses while highlighting the unique role of structural disorder in ensuring an efficient replication and transcription of the paramyxoviral genome. We accordingly discuss through the manuscript the functional role of induced folding and residual flexibility in terms of transcription, replication and molecular partnership. We also underscore how targeting the N–P interaction holds promises for new antiviral approaches. We finally conclude by highlighting the functional implications and general advantages of structural disorder within viruses.

## 2. Abundance of Structural Disorder in Paramyxoviral N and P Proteins

### 2.1. Paramyxoviral P Proteins Are Highly Disordered

The P gene of *Paramyxovirinae* members illustrates how a virus encodes as much information as possible in a single gene. Indeed, the *P* gene can give rise to a number of different polypeptide products by means of either overlapping reading frames, or of a peculiar transcription process whereby one or more non-templated nucleotides are inserted, resulting in a shift of the reading frame during translation. Accordingly, the P genes encode at least two non-structural proteins (C and V) in addition to the P protein.

**Figure 2:**
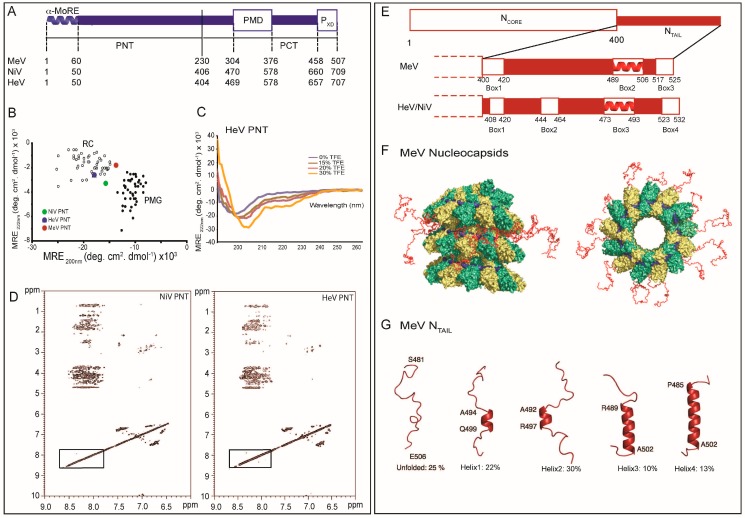
Structural disorder within paramyxoviral N and P proteins. (**A**) Modular organization of the MeV, NiV and HeV P proteins. Domain organization of P showing that it is composed in the three viruses of two moieties, PNT and PCT, that are separated by a vertical solid line. Dotted lines indicate the borders of the different domains in the three viruses. Structured and disordered regions are represented as large or narrow boxes, respectively. PNT: N-terminal region of P; PCT: C-terminal region of P; PMD: P multimerization domain; P_XD_: X domain of P. The α-MoRE at the N-terminus of PNT, which is partly preconfigured in solution as an α-helix, is shown; (**B**) 222–200 nm ellipticity plot (modified from [27]). The mean residue ellipticity values at 222 nm of a set of well-characterized unfolded or pre-molten proteins have been plotted against the mean residue ellipticity values at 200 nm. The position in the plot of MeV and *Henipavirus* PNT is highlighted. RC: random coil; PMG: premolten globule-like. Note that HeV PNT is the most extended domain; (**C**) Far-UV circular dichroism spectra of HeV PNT in the absence and the presence of increasing concentrations of TFE (15%, 20% and 30%) showing that HeV PNT is able to fold into an α-helical conformation; Data were taken from [27] (**D**); Two-dimensional ^1^H NMR NOESY spectra of *Henipavirus* PNT proteins (modified from [30]); (**E**) Modular organization of the MeV, NiV and HeV N proteins. Domain organization of N showing that it is composed in the three viruses of two moieties, N_CORE_ that is ordered and N_TAIL_ that is disordered. Ordered and disordered regions are represented as large or narrow boxes, respectively. The N_TAIL_ region from the three viruses is zoomed out to show the various boxes that correspond to putative or experimentally proven MoREs. The α-MoRE that is involved in the interaction with the P_XD_ is shown as a red helix; (**F**) NMR-based model to locate MeV N_TAIL_ within the viral nucleocapsid. N_CORE_ monomers are colored in green and yellow, while the RNA is shown in blue. Front (**left**) and top (**right**) views of 13 N_TAIL_ conformers (shown in red) sampling an ensemble of conformations that point out from the surface of the viral nucleocapsid. (Adapted with permission from [46]). Copyright 2011 National Academy of Sciences; (**G**) Quantitative ensemble description of MeV N_TAIL_ in isolation. Four conformational states of the intrinsically disordered N_TAIL_, as derived by NMR spectroscopy, are represented by cartoon structures. Adapted with permission from [46]). Copyright 2011 National Academy of Sciences.

Although paramyxoviruses share a similar P modular organization, *Henipavirus* P proteins (707 amino acids in HeV and 709 amino acids in NiV), are much larger than those of other paramyxoviruses (507 amino acids in MeV) (Figure 2A) [3]. In all cases, computational analyses showed that the P proteins consist of an N-terminal domain (PNT) that is predicted to be intrinsically disordered (Figure 2B) and is also found in the V protein, and a C-terminal domain (PCT) that can be further subdivided in various regions [30,31]. The disordered state of MeV, NiV and HeV PNT was confirmed using a wide range of biochemical and biophysical approaches. Indeed, PNT domains were found (i) to be highly sensitive to proteolysis; (ii) To possess NOESY and circular dichroism (CD) spectra typical of IDPs (Figure 2C,D); (iii) To possess Stokes radii (R_S_) much larger than those expected for globular proteins with the same size and (iv) to gain structure upon addition of the secondary structure stabilizer 2,2,2-trifluoroethanol (TFE) (Figure 2C) [30,31,32]. Beyond PNT, other IDRs occur within the P protein. In fact, PCT is composed of alternating disordered and ordered regions (Figure 2A) [30,32]. As a result, as much as 70%–80% of the residues in MeV, NiV and HeV P proteins are disordered. Amongst the ordered domains in PCT are the P multimerisation domain (PMD) and the C-terminal X domain (P_XD_), which are responsible respectively for the oligomerization of P and for binding to the C-terminal domain of N (*i.e.*, N_TAIL_) [33,34,35,36,37]. PMD and P_XD_ are separated by a flexible linker region predicted to be poorly ordered [31]. Indeed, in the case of SeV, NMR studies carried out on the C-terminal region of P showed that the region upstream P_XD_ is disordered [38,39,40]. Besides, an additional flexible region (referred to as “spacer”) occurs upstream PMD in MeV, NiV and HeV [30,31,41].

*Paramyxoviridae* P proteins are phosphorylated at multiple sites, with these phosphorylation sites being interestingly located within the disordered PNT domains [31,42,43,44]. This is in good agreement with the findings by Iakoucheva *et al.* [45], pointing out the importance of structural disorder for post-translational modifications (PTMs) such as phosphorylation. Indeed, PTM of proteins has three structural requirements: an appropriate local sequence, structural exposure, and flexibility of the site so that it can be productively accommodated within the active site of the modifying enzyme. These requirements are in an intimate relationship with structural disorder.

### 2.2. Paramyxoviral N Proteins Self-Assemble into Nucleocapsid-Like Structures from Which Protrude Disordered Tails

N proteins from *Paramyxovirinae* members are proteins of more than 500 amino acids in length, which are responsible for encapsidating the viral RNA. The resulting ribonucleoproteic (RNP) complex has a typical “herringbone”-like structure as seen in electron micrographs of paramyxoviruses [6,7]. In the *Paramyxovirinae* subfamily, each N protein covers six nucleotides, a feature that explains the so-called “rule of six”, *i.e.*, the requirement for the viral genome to be a multiple of six in order to ensure efficient transcription and replication. In infected cells, N binds only to genomic RNAs and not to cellular RNA or viral mRNAs. However, when expressed in heterologous systems in the absence of other viral proteins, recombinant nucleoproteins bind cellular RNAs and form nucleocapsid-like structures that are almost indistinguishable from viral nucleocapsids [6,47,48,49]. Electron microscopy (EM) studies and dynamic light scattering (DLS) measurements of recombinant N proteins revealed that they are highly polydisperse and have a broad size distribution, with nucleocapsid-like particles having been found to adopt different forms, ranging from spherical, to ring-like and herringbone-like [7,50].

Deletion analysis, EM and NMR studies showed that *Paramyxoviridae* N proteins are divided into two regions: a structured N-terminal moiety, N_CORE_, well conserved in sequence and which contains all the regions necessary for self-assembly and RNA-binding, and a C-terminal domain, N_TAIL_ (Figure 2E) (for reviews see [24,26,27,51]). N_TAIL_ protrudes from the globular body of N_CORE_ and is exposed at the surface of the viral nucleocapsid [46,52,53,54]. N_TAIL_ contains the regions responsible for binding to P in both N°-P and N^NUC^-P complexes [34,36,37,51,55,56,57]. EM analysis highlighted a cross-talk between N_CORE_ and N_TAIL_, as judged based on the observation that removal of the disordered N_TAIL_ domain leads to increased nucleocapsid rigidity, with significant changes in both pitch and twist [57,58,59].

N_TAIL_ domains possess features that are hallmarks of intrinsic disorder: (i) Hyper-sensitivity to proteolysis [30,54]; (ii) Absence of corresponding density in cryo-EM reconstructions of nucleocapsids [58]; and (iii) High variability in the amino acid sequences amongst phylogenetically related members. In addition, the sequence properties of N_TAIL_ conform to those of IDPs being enriched in polar and charged residues and depleted in hydrophobic residues. Hydrodynamic and spectroscopic analysis (*i.e.*, size exclusion chromatography, SEC, DLS, CD and NMR) confirmed the disordered state of N_TAIL_ unveiling that roughly 25% of paramyxoviral N sequences are in the unfolded state (for a review see [26]).

High-resolution structural data on *Paramyxoviridae* N is limited. So far, the only crystal structures of N proteins that have been solved are those of the N protein from RSV and from parainfluenza virus 5 (PIV5, a *Rubulavirus* member within the *Paramyxovirinae* subfamily) [60,61]. In both cases, N has been solved in the form of recombinant N-RNA rings. Indeed, following heterologous expression, the N proteins of these viruses bind to short cellular RNAs and form short N-RNA complexes that close up into rings through protein-protein contacts. Such rings correspond to one turn of a nucleocapsid helix, although they have limited flexibility due to sterical constraints [47]. While in the case of PIV5, the N protein was subjected to limited proteolysis to remove the N_TAIL_ disordered region prior to crystallization [61], in the case of RSV, it is the full-length form that was crystallized [60], in line with the fact that the RSV N protein is shorter than its *Paramyxovirinae* counterparts and devoid of the disordered N_TAIL_ region. In both RSV and PIV5, the nucleoprotein consists of two lobes (NTD and CTD) separated by a hinge that accommodates the RNA. The RNA is tightly packed between the two N lobes, being located on the external face of N:RNA rings [60,61]. Each N protomer contacts 6 (PIV5) or 7 (RSV) nucleotides. For both RSV and PIV5, each protomer in the N-RNA ring makes extensive contacts with its neighbors through its C-terminal and N-terminal domain: each N subunit possesses an extended N-terminal and C-terminal arm (NTD-arm, CTD-arm) that makes contacts with the preceding (N_i − 1_) and following (N_i + 1_) protomer, respectively. By taking advantage of the predicted structural similarity between RSV and MeV N, the atomic model of the RSV N monomer in N-RNA rings was used as template to generate a model of MeV N:RNA that was docked within the electron density map of MeV nucleocapsids [62]. Although the disordered N_TAIL_ domain could not be resolved in the reconstruction of the nucleocapsid, the fit suggests that N_TAIL_ points toward the interior of the helical nucleocapsid [62]. Ringkjobing-Jensen and colleagues proposed a NMR and SANS-based model that gave a structural framework for understanding the role of MeV N_TAIL_ in nucleocapsids (Figure 2F) [46]. In this model, the first 50 residues of N_TAIL_ point indeed towards the helix interior and form an articulated spacer that allows the remainder of the chain, encompassing the P_XD_ binding site, to escape from the interior of the capsid via the confined interstitial space between successive turns of the capsid helix while retaining a high conformational freedom [46]. Importantly, these studies proved the disordered state of MeV N_TAIL_ not only in isolation, but also in the context of intact nucleocapsids. Later on, using a similar NMR approach, experimental evidence was obtained supporting that N_TAIL_ domains from both NiV and HeV retain their disordered state *in situ*, *i.e.*, when appended to nucleocapsids [63,64]. Like in the case of MeV, the resonance behavior observed with *Henipavirus* nucleocapsids, supports a model in which the first 50 disordered amino acids of N_TAIL_ are conformationally restricted. Notably, this model provides a plausible explanation for the increased rigidity of MeV nucleocapsids in which the flexible N_TAIL_ region has been cleaved off [57,59,65]. Accordingly, one could speculate that the inherent flexibility of the N_TAIL_ region in sandwich between successive turns of the nucleocapsid can be the basis for variations in pitch and twist that can be related to switches between transcription and replication [49].

The crystal structure of a monomeric, RNA-free form of the NiV N protein devoid of the NTD-arm and of N_TAIL_ in complex with the N-terminal N°-binding region of P (P_NTD_, amino acids 1–50 of P) has also been solved [66]. P_NTD_ binds to CTD and interferes with the binding of the CTD-arm from the N_*i* −__1_ protomer and the NTD-arm from the N_*i* + 1_ protomer thereby providing a structural explanation for the ability of PNT to prevent N self-assembly [66].

Recently, elegant cryo-EM studies led to almost atomic resolution of the MeV helical nucleocapsid formed by the folded N_CORE_ domain [65]. Combined with the atomic structures of RSV N and NiV N°_CORE_−P_NTD_, 3D reconstruction of MeV helical nucleocapsid allowed building a reliable pseudo-atomic model of the MeV N_CORE_-RNA helix. Those studies confirmed the role of the NTD-arm and CTD-arm in maintaining the cohesion of N protomers and in rigidifying the CTD thus keeping N in a closed conformation allowing the RNA to be trapped [66].

All structural data gathered so far indicate that in *Paramyxoviridae* nucleocapsids the RNA is not accessible to the solvent, and has to be partially released from N to become accessible to the polymerase. Therefore, a conformational change must occur within N to allow exposure of the RNA. The disordered N_TAIL_ domain is thought to play a major role in this conformational change.

### 2.3. Folding Propensities of the Disordered N_TAIL_ and PNT Domains

Although N_TAIL_ and PNT are mostly unfolded in solution, they have been shown to retain some degree of compaction based upon their Stokes radii (R_S_) and their ellipticity values at 200 and 222 nm, which are consistent with a PMG state [26,32,57]. PMGs are characterized by a conformational state between the RC and the MG state, and possess a certain degree of compaction due to the presence of fluctuating secondary and/or tertiary structures. Indeed, the addition of urea increased the R_S_ of both N_TAIL_ and PNT supporting a conformational state that is not completely unfolded in solution [30].

In general, PMGs possess more hydrophobic residues than RCs. This is readily appreciable in the HCA plots of N_TAIL_ and PNT that are characterized by the presence of short regions locally enriched in hydrophobic clusters (see Figure 2 and Figure 3 in [26]). These regions correspond to putative Molecular Recognition Elements (MoREs). MoREs are short, order-prone regions within IDPs that have a certain propensity to bind to a partner and thereby to undergo induced folding (*i.e.*, a disorder-to-order transition) [67,68,69,70]. In fact, it is currently accepted that the crux of molecular recognition by IDPs is ensured by MoREs. This phenomenon could be explained by the fact that MoREs in the PMG state present a significant interest from an energetic point of view as they facilitate the folding upon binding process [70]. In other words, the residual structure restrains the conformational space sampled by IDPs, thereby reducing the number of interconverting conformers in solution and rendering the structural transition of the IDP to the (partially) folded conformation energetically less demanding.

In the case of PNT, predictions pointed out the presence of a short (40–50 amino acids) order-prone segment at its N-terminus (Figure 2A). This N-terminal module with α-helical propensity corresponds to a conserved region amongst *Avulavirus* and *Rubulavirus* members [31]. The involvement of the N-terminal PNT region in N°-binding has been experimentally confirmed in the case of rubulaviruses and of NiV [66,71]. Using computational approaches, it has been shown that all *Paramyxovirinae* P proteins share a short (11–16 residues) sequence motif within their first 40 residues [72]. It has been proposed that this region would be conserved in all *Mononegavirales* P proteins as a result of divergent evolution, and would be involved in binding to N° [72]. In agreement, a similar N-terminal module, globally disordered yet containing transient α-helices (aa 1–60) has also been identified and characterized in the P protein from the vesicular stomatitis virus (a rhabdovirus) [73], and subsequently shown to fold upon binding to N° [74]. The N-terminal region of *Paramyxovirinae* P likely corresponds to an α-helical MoRE (α-MoRE). The folding potential of MeV, NiV and HeV PNT domains was also confirmed by far-UV CD studies, where increasing concentrations of TFE were shown to induce a pronounced gain of α-helicity [30,32]. In addition, in the case of MeV PNT, limited proteolysis experiments in the presence of TFE led to the identification of a thermolysin-resistant fragment. This fragment, spanning residues 27–99, contains a protein region (aa 27–38) with a strong propensity to fold as an α-helix. The extent of residual compaction within the three PNT domains follows the order NiV PNT > HeV PNT > MeV PNT (Figure 2B). Although it is more than plausible that the N-terminal region of MeV and HeV PNT folds upon binding to N°, assessment of the effective folding upon binding abilities of this putative α-MoRE awaits however the isolation and purification of a binding partner. Beyond N°, one such a possible binding partner can be L and/or SNAP29, by analogy with the closely related RDV and human parainfluenza type 3 virus, respectively [75,76].

**Figure 3:**
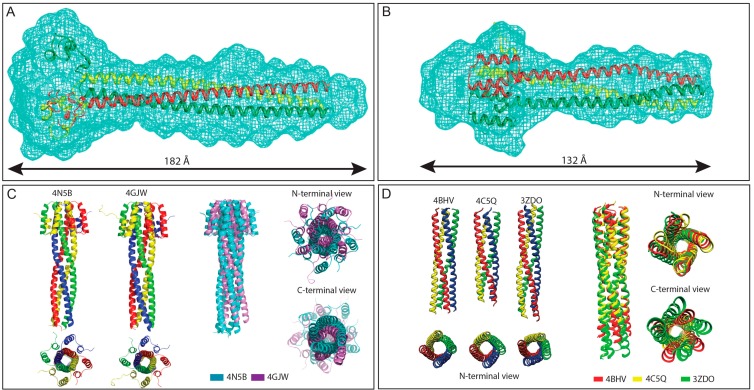
Structures of *Henipavirus* and MeV P multimerization domains. (**A**,**B**) SAXS-derived *ab initio* envelopes of NiV (**A**) and HeV (**B**) PMD; The structures of a trimeric coiled-coil model of NiV (**A**) and HeV (**B**) PMD are shown embedded in their respective envelope, with the three chains being displayed in three different colors. Data from panel A and B were taken from [77,78], respectively; (**C**) Structural comparison among NiV PMD crystal structures. **Left**: Ribbon model of the two crystal structures of NiV PMD solved so far (pdb codes 4N5B and 4GJW), with the four chains being displayed in four different colors; **Right**: superimposition of the two NiV PMD tetramers, with PDB entries 4N5B and 4GJW shown in cyan and violet, respectively; and (**D**) Structural comparison among MeV PMD structures. **Left**: ribbon representations of the crystal structures of the MeV PMD tetramers as observed in the three different MeV PMD forms solved to date; **Right**: superimposition of the three MeV PMD tetramers, with PDB entries 4BHV, 4C5Q and 3ZDO shown in red, yellow and green, respectively. Data from panel (**C**) were taken from [79] and were reproduced with permission of the International Union of Crystallography.

In the case of N_TAIL_ domains, the structural properties of the MoREs have been unraveled using conformational and spectroscopic analyses. For both NiV and HeV, the latter analyses have been applied on full and truncated forms of the N_TAIL_ regions bearing various combinations of the four predicted MoREs (Figure 2E) [80]. The firth and fourth MoREs (amino acids 408–422 and 523–532, respectively) were found to possess irregular forms (*i.e.*, I-MoRE), while the MoREs encompassing residues 444–464 (*i.e.*, Box2) and 473-493 (*i.e.*, Box3) were found to have clear α-helical propensities (*i.e.*, α-MoREs) [80]. These results contrast the sequence-based secondary structure predictions that pointed out the occurrence of an α-helix within Box1 and of a β-strand within Box2 [30]. However, it has been frequently reported that some MoREs possess ambiguous amino acid sequences thus misleading secondary structure predictions [81]. In addition, some IDPs possess the capacity to adapt to the structure of distinct partners [82]. Whether these erroneous predictions of the secondary structure content of Box1 and Box2 reflect an intrinsic limitation of the predictors or rather intrinsic plasticity of these Boxes allowing them to adopt different conformations in a template-dependent manner remains to be unraveled and awaits the identification of their corresponding partners. Those studies also showed that Box1 is a major determinant of protein compaction and Box4 is likely involved in the stabilization of the α-helices located in Box2 and Box3 [80]. Furthermore, site-directed spin-labeling (SDSL) EPR spectroscopy studies unveiled a considerable conformational heterogeneity within Box3, consistent with the occurrence of multiple helical conformers of different length [83]. In agreement, analysis of the Cα chemical shifts of the free form of HeV and NiV N_TAIL_ domains showed that Box3 is at least transiently populated as an α-helix, and in the case of NiV, a second, short α-helical region could be detected within Box2 [63,64].

In the case of MeV N_TAIL_, an α-MoRE (aa 488–499 of N) has been identified within one (namely Box2) out of three regions conserved in *Morbillivirus* members (Figure 2E). The role of this α-MoRE in binding to P_XD_ and in the α-helical induced folding of N_TAIL_ was confirmed by spectroscopic and biochemical experiments carried out on a truncated N_TAIL_ form devoid of the 489–525 region [33] and subsequently by structural studies [36]. Interestingly, using MD simulations, the isolated α-MoRE was found to behave like a MG [84]. Indeed, the distribution of the radius of gyration (R_g_) of the α-MoRE was found to be smaller in the unbound state than in the bound state, indicating that the majority of the conformations of the free form of the α-MoRE are more compact than the bound conformation (the folded state). Analysis of the Cα chemical shifts of N_TAIL_ and of the mobility of spin labels grafted within Box2 showed that the α-MoRE of MeV N_TAIL_ is partly preconfigured as an α-helix in the absence of P_XD_ [85,86,87]. More recently, an atomic-resolution ensemble description of the α-MoRE of MeV N_TAIL_ could be obtained using recently developed tools designed to provide quantitative descriptions of conformational equilibria in IDPs on the basis of experimental NMR data (Figure 2G) [38,88]. By combining residual dipolar couplings (RDCs) measurements and ensemble optimization methods [38,88], the α-MoRE was shown to exist in a rapidly interconverting conformational equilibrium between an unfolded form and conformers containing four discrete α-helical elements situated around the interaction site [46]. All of these α-helices are stabilized by N-capping interactions mediated by side chains of four different aspartic acids or serines that precede the observed helices [89]. N-capping stabilization of helices or turns represents an important mechanism by which the primary sequence encodes pre-recognition states, and has already been observed in other IDPs including SeV N_TAIL_ [88]. In this latter case, the α-MoRE was shown to possess a similar conformational behavior, although in this case the α-MoRE samples an extended conformation and only three helical conformers [51,88].

Comparison between NiV, HeV and MeV N_TAIL_ protein sequences pointed out the occurrence of an additional MoRE in the case of *Henipavirus* N_TAIL_ (*i.e.*, Box2), where the Box3 region corresponds functionally to Box2 of MeV N_TAIL_ based on binding abilities towards P_XD_ (Figure 2E) [35,36,80,90]. This finding further emphasizes the plasticity of IDP/Rs, which tolerates insertions/deletions in functionally relevant regions.

N_TAIL_ and PNT domains are disordered not only in isolation but also within the full-length N and P proteins. Indeed, N and P proteins have been found to be highly sensitive to proteolysis [90] (Beltrandi, Habchi, Longhi and Cavalli, unpublished data). In addition, as already mentioned, many detectable peaks in the Heteronuclear Single Quantum Correlation (HSQC) spectra of intact nucleocapsids well superimpose onto those of the free N_TAIL_ regions indicating that N_TAIL_ is disordered within nucleocapsids-like particles [46,63,64].

From a functional point of view, beyond imposing structural constraints to the conformational ensemble of IDPs, the main function of MoREs is to allow engagement in a broad molecular partnership. Indeed, in the case of MeV, the flexible N_TAIL_ domain has been shown to bind via its MoRE to numerous partners including the X domain of the P protein [33,35,57,91], the major inducible heat shock protein hsp70 [92,93,94], the interferon regulatory factor 3 [95,96], a yet unidentified protein cell receptor involved in MeV-induced immunosuppression [97,98], a nuclear export protein [99], the matrix protein [100], peroxiredoxin 1 [101] and possibly, components of the cell cytoskeleton [102,103]. In the case of NiV and HeV, although only one N_TAIL_ partner has been identified and characterized (*i.e.*, P_XD_) so far, an even broader molecular partnership is expected with respect to MeV N_TAIL_, based on the presence of the additional MoRE (*i.e.*, Box2). Amongst all the interactions established by N_TAIL_, the interaction with P_XD_ is critical as it allows the P/L complex to be recruited onto the nucleocapsid in order to allow transcription and replication to take place [22,23,25]. In striking contrast, a recent study by the group of Plemper has challenged the well-established model according to which Box2 is strictly required to recruit the MeV polymerase complex: indeed, Box2 was found to be dispensable for MeV transcription and replication in the absence of the upstream N_TAIL_ region that was found to act as a negative modulator (*i.e.*, to prevent binding of the L–P complex to the nucleocapsid) [104]. Similarly to N_TAIL_, PNT domains have been reported to interact with multiple partners, including N in both assembled and unassembled forms [105], cellular proteins [106] and the L protein [107,108].

## 3. Differential Compaction and Flexibility within Coiled-Coils of Paramyxoviral Phosphoproteins

Sequence analyses predict a coiled-coil region within *Paramyxoviridae* PMD [30,31]. The coiled-coil organization has been experimentally confirmed in the case of SeV [109], RDV [110], MeV [41,79], RSV [111], mumps virus (MuV, a *Rubulavirus*) [112] and human metapneumovirus (a *Pneumovirinae* member) [113]. In all cases, the P proteins were shown to oligomerize, with oligomerization being a prerequisite for the so-called “cartwheeling” mechanism of the viral polymerase movement that posits that the L protein progresses from one N protomer to the following in order to ensure transcription and replication. Until recently, structural data gathered on PMDs over the last decade consistently converged to show that paramyxoviral P proteins possess a tetrameric coiled-coil organization characterized by a parallel orientation except for MuV P, where the tetramer was found to consist of two sets of antiparallel helical dimers [112]. The tetrameric organization of paramyxoviral PMDs has been however challenged recently by the finding that *Henipavirus* PMDs adopt a trimeric organization in solution [77,78] (Figure 3A,B). Surprisingly however, NiV PMD was found to adopt a tetrameric organization in crystals [114] (Figure 3C). The trimeric state of *Henipavirus* PMDs was confirmed by several independent biochemical and biophysical approaches, including SEC, SDS-PAGE, cross-linking, analytical ultracentrifugation and SAXS [77,78]. Interestingly, recent SAXS studies confirmed the trimeric state of HeV PMD within the entire PCT region, thus extending and strengthening the conclusions based on PMD [78]. The shape of the HeV PMD SAXS envelope was found however to be different from that of NiV PMD, with HeV PMD displaying a much smaller R_g_ and D_max_. This difference has been ascribed to a different orientation of the N-terminal helical region (referred to as “head”) of PMD: while in the case of NiV the head is in the up orientation being exposed to the solvent, it adopts a down orientation being packed back against to coiled-coil in the case of HeV (Figure 3A,B) [78]. Given the high sequence similarity between NiV and HeV PMDs, these discrepancies were taken to reflect the ability of *Henipavirus* PMDs to undergo conformational changes resulting in forms of different lengths and compaction, with these different forms being possibly related to the various functions of P during the viral cycle [78].

A possible explanation for the observed discrepancies between the solution and crystal conformations of NiV PMDs, is the high local protein concentrations and/or the strong inter-molecular interactions within crystals that might have biased the oligomeric state of the protein and promoted a tetrameric organization. In any case however, it is already established that coiled-coils are able to modulate their oligomeric state according to the physico-chemical conditions (pH, temperature) or depending on whether they are located inside or outside the cell [115,116]. Interestingly, the GCN4 leucine-zipper domain was shown to adopt different oligomeric states depending on the crystallization conditions, implying that the amino acid sequence does not specify a unique oligomeric state [117]. It is also worthy to emphasize that conflicting experimental evidence are not unique to NiV PMD: indeed SeV PMD had also been shown to form trimers in solution [118,119] and to adopt a tetrameric coiled-coil conformation in crystals [109]. The experimental evidence supporting a trimeric state of SeV P have been neglected perhaps too rapidly in light of the crystallographic data pointing to a tetrameric organization. However, the finding that both SeV and NiV PMD can form trimers in solution and tetramers in crystals may reflect their intrinsic ability to adopt different oligomeric states that could be related to different functional forms of the P protein and to the different complexes (*i.e.*, N–P, N°–P, P–L) that it can form within infected cells.

In the same vein, structural comparison amongst the different crystallographic structures of MeV PMD solved so far unveiled unexpected structural variations [41,79]. Although all the structures have a tetrameric coiled-coil organization, structural comparison unveiled considerable differences not only in the quaternary structure but also in the extent of disorder within the C-terminal region of the coiled-coil (Figure 3D). The disordered nature of the C-terminal region is also supported by SAXS and SEC studies, where the latter show that MeV PMD exists as a dynamic equilibrium between two tetrameric forms of different compaction [79]. Of note, structural comparison between the two crystal structures of NiV PMD solved so far (pdb codes 4N5B and 4GJW), revealed a similar extent of structural polymorphism with notable differences in the quaternary structure and in the amount of disorder at the C-terminal end. Strikingly, for both MeV and NiV, no disorder-associated sequence feature is discernible in the C-terminal region of their PMD, which well exemplifies how disorder cannot be always anticipated.

The unexpected plasticity and flexibility of both MeV and NiV PMD could be a hint of the existence of different functional forms of the P protein reflecting its multifunctional nature and pivotal role in the replicative cycle. These results also unveiled that coiled-coils structure can exhibit a certain degree of freedom, and that coiled-coils are less rigid than previously thought. They also illustrate how conclusions about function and mechanism based on analysis of a single crystal structure of a dynamic protein can be easily biased, and they challenge to some extent the assumption according to which coiled-coil structures can be reliably predicted from the amino acid sequence [79].

In conclusion, the ability of SeV, NiV and HeV PMD to adopt different oligomeric states, together with the ability of MeV PMD to dynamically sample different forms differing in the degree of compaction and in the extent of disorder, might be the basis for the ability of P to form different complexes critical for transcription and replication, with conformational changes possibly dictating the ability to form a transcriptase *vs.* a replicase complex. Additional studies are necessary to obtain definite answers as to whether P oligomerization is strictly required for transcription and replication. Likewise, detailed understanding of the role of disorder within PMD and of the functional impact of varying the P oligomeric state awaits future mutational studies.

## 4. Molecular Mechanisms of Paramyxoviral N_TAIL_–P_XD_ Interactions: A Continuum of Disorder from the Free to the Bound Form

### 4.1. Molecular Polymorphism in N_TAIL_–P_XD_ Interactions

**Figure 4:**
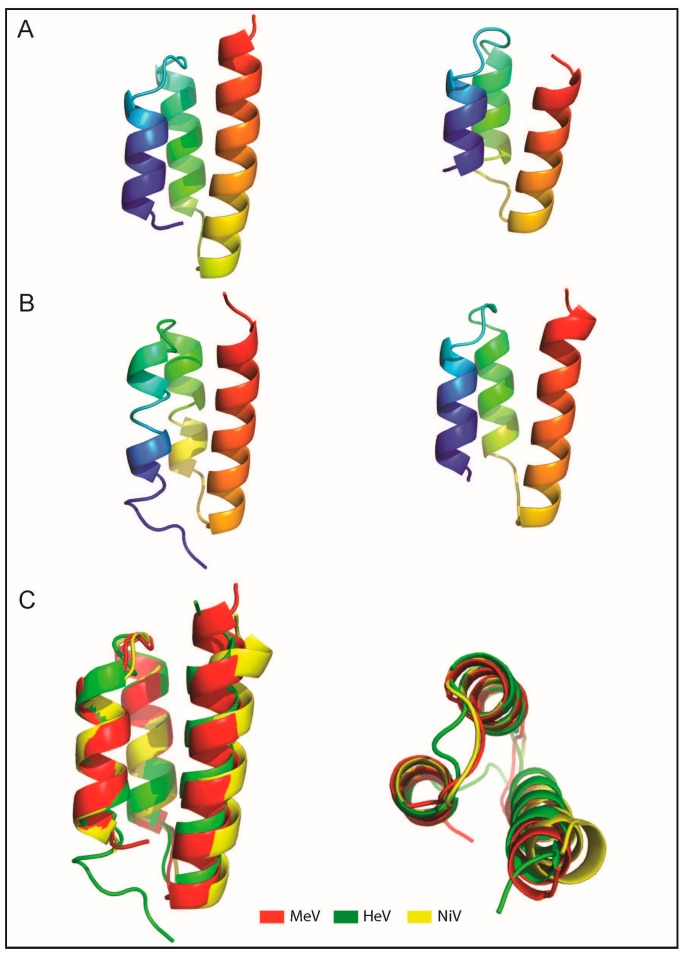
Structures of MeV and *Henipavirus* X domains. (**A**) Ribbon representation of the crystal (**left**) and solution (**right**) structure of MeV P_XD_ (pdb codes 1OKS and 2K9D); (**B**) Ribbon representation of the crystal structure of HeV P_XD_ (**left**, pdb code 4HEO) and of a model of NiV P_XD_ (**right**) [90]; (**C**) Superimposition of the crystal structures of MeV and HeV P_XD_ onto the NiV P_XD_ model. Structures were drawn using Chimera [123] and Pymol [124].

As already discussed above, the P protein simultaneously binds to L and to the exposed C-terminal domain of N (N_TAIL_) via its C-terminal X domain (P_XD_). The structures of MeV and HeV P_XD_ have been solved and were shown to consist of an anti-parallel triple α-helical bundle (Figure 4A,B). The surface of P_XD_ formed between helices α2 and α3 displays a large hydrophobic cleft [35,37,63,87]. High-resolution structural data are also available for the X domains of the closely related SeV and MuV viruses, the structures of which have been solved by NMR and X-ray crystallography, respectively [39,120]. Interestingly, although the structure of SeV P_XD_ resembles that of MeV P_XD_, in that it is also formed by three α-helices folded around a hydrophobic core [39], negatively charged residues dominate the surface created by α2 and α3 helices [34]. Another interesting feature is that whilst the fold of MuV P_XD_ is conserved (*i.e.*, three α-helices), it actually exists as a MG in solution as evidenced by CD, NMR and DLS experiments [120]. The observed stable 3D structure in crystals of MuV P_XD_ apparently results from a stabilizing effect brought by the addition of methylamine cosolute during crystallogenesis experiments [120]. Lack of a unique stable 3D structure is not a feature unique to MuV P_XD_ being also shared by the corresponding domains from other rubulaviruses that were found to span a structural continuum ranging from stable α-helical bundles to largely disordered forms in solution [121]. In the same vein, recent electron spray ionization mass spectrometry (ESI–MS) studies unveiled that MeV P_XD_ has a bimodal charge state distribution reflecting the presence of two forms differing in the extent of compaction [122].

Although the structure of NiV P_XD_ has not been solved yet, it is expected to adopt a structure similar to that of its HeV counterpart (Figure 4B) based not only on the high sequence similarity between the two domains (94%), but also on their common spectroscopic features. Indeed, HeV and NiV P_XD_ possess similar CD and NOESY spectra typical of α-helical folded domains [90]. Thus the structural fold of the P X domains is conserved within paramyxoviruses, suggesting that their function is conserved as well (Figure 4C).

Both NiV and HeV P_XD_ form with N_TAIL_ a 1:1 stoichiometric complex that is stable up to 1 M NaCl, and whose K_D_ is in the μM range [90]. The α-MoRE spanning residues 473–493 (*i.e.*, Box3) has been shown to be the P_XD_ binding site [80]. CD, NMR and SDSL EPR studies showed that *Henipavirus* N_TAIL_ domains undergo P_XD_-induced α-helical folding within Box3 with the remainder of the chains remaining in the disordered state [63,64,80,90]. Interestingly, the conformational ensemble that is sampled by the free form of N_TAIL_ in solution presages the α-helical conformation of the P_XD_-bound form, as judged from chemical shift analysis [63,64].

Although direct structural data on *Henipavirus* N_TAIL_–P_XD_ complexes are still lacking, recent NMR studies provided first clues on the structure of these complexes. In particular, the vanishing of the resonances of the α-MoRE upon addition of P_XD_ supports the existence of a highly dynamic complex for both NiV and HeV, with the α-MoRE undergoing α-helical fraying at the surface of P_XD_ [63,64,90]. In spite of these similarities, subtle differences distinguish the two *Henipavirus* N_TAIL_–P_XD_ complexes. Indeed, the NiV N_TAIL_–P_XD_ complex is slightly tighter than that of HeV [63,64,80,90], in line with the ability of SEC to document complex formation in the case of NiV but not in the case of HeV [90]. Besides, the α-MoRE region within NiV N_TAIL_ was found to possess a higher degree of pre-configuration with respect to HeV N_TAIL_, as judged based on the ^15^N R_2_ values [64], and to populate longer-lived interconverting α-helical segments that could be detected by EPR measurements [83]. Furthermore, NMR experiments showed in the case of NiV N_TAIL_ an additional involvement of Box2 in the interaction with P_XD_ [64]. That Box2 does participate to some extent to NiV N_TAIL_ binding to P_XD_ was also documented by SDSL EPR measurements showing that in the case of NiV, but not of HeV, the addition of the partner triggers a reduction in the mobility of a spin label grafted within Box2 [83]. The differences that were observed between NiV and HeV N_TAIL_ regions in their free and bound forms, in particular within Box2, might be dictated by a substitution occurring within Box2 at position 457, where an Asp residue in HeV N_TAIL_ is replaced by an Asn in NiV N_TAIL_.

In the case of HeV, analysis of chemical shift perturbations in reciprocal titration studies and the availability of the crystal structure of P_XD_ allowed identification of the residues involved in the interaction [63]. Those studies revealed that although the binding interface is made of hydrophobic residues, the binding pocket of P_XD_ is surrounded by charged residues that may establish electrostatic interactions with basic residues of Box3. Subsequent isothermal titration calorimetry (ITC) studies carried out at different pH values did in fact confirm the role of electrostatics in complex formation, a conclusion further strengthened by mutational studies that targeted charged residues both within N_TAIL_ and P_XD_ [125]. Collectively, those studies provided direct evidence that charged residues surrounding the hydrophobic binding interface play a crucial role in complex formation, thus arguing for a multiparametric interaction and emphasizing the role of residues located in the neighborhood of the binding interface. Accordingly, it has been proposed that the HeV N_TAIL_/P_XD_ complex formation may rely on the so-called “electrostatic steering mechanism” [126], where long-range electrostatic forces pull N_TAIL_ toward the relevant acidic patch on the surface of P_XD_, thus leading to an “electrostatic encounter complex” [127] in which N_TAIL_ is loosely anchored at the periphery of the binding site [125].

The corollary of this model is that HeV N_TAIL_ would fold after binding, a behavior corroborated by quantitative analysis of NMR titration data (see below) [63].

Taking into account the role of electrostatics in the formation of the HeV N_TAIL_–P_XD_ complex, it is conceivable that the Asp to Asn substitution could be responsible for the observed differences in the role of Box2 in binding to P_XD_ by NiV and HeV N_TAIL_.

Neither chemical shifts nor electrostatic interactions are able to distinguish rotational symmetry about the axis of the N_TAIL_ helix, although two conformations are most probable, both having the hydrophobic face of the α-MoRE in contact with the hydrophobic interface of P_XD_. Through a combination of mutational and SAXS studies, experimental evidence was recently gathered supporting a parallel orientation of the MoRE at the surface of HeV P_XD_ [125].

**Figure 5:**
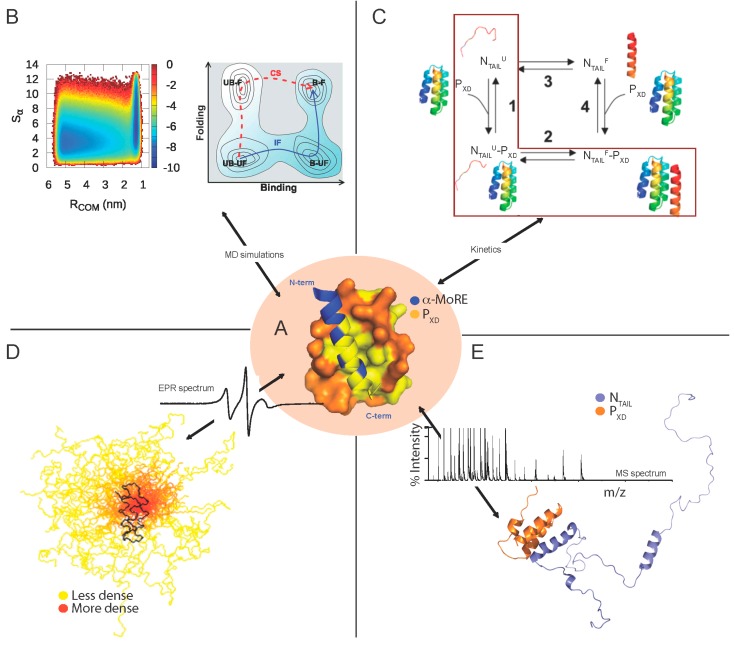
Molecular mechanisms and fuzziness of the MeV N_TAIL_–P_XD_ complex as unveiled by independent experimental evidences. (**A**) Structure of the MeV chimeric N_TAIL_–P_XD_ construct (PDB code 1T6O) [36]. MeV P_XD_ (amino acids 459–507 of P) is shown in orange with surface presentation while the α-MoRE region (amino acids 486–504 of N) is shown in blue with ribbon representation. Hydrophobic residues are shown in yellow; (**B**) (**Left**) Free-energy surface for the interaction between the α-MoRE and MeV P_XD_ as a function of R_COM_ (binding order parameter) and Sα (folding order parameter); (**Right**) Schematic free-energy surface showing that folding upon binding takes place according to an induced folding (IF) mechanism and not to a conformational selection (CS) mechanism despite the preexistence of unbound-folded state (UB-F). Note that, in the folding upon binding process, there are four possible states: unbound-unfolded (UB-UF), bound-unfolded (B-UF), unbound-folded (UB-F), and bound-folded (B-F). The schematic picture gives a good illustration that the preexistence of the UB-F state is a necessary but not a sufficient condition for a conformational selection mechanism. (Adapted with permission from [84]). Copyright 2013 National Academy of Sciences; (**C**) A kinetic-based model showing the folding after binding mechanism of the MeV N_TAIL_–P_XD_ interaction (highlighted in a red square). Indeed, N_TAIL_ recognizes P_XD_ by first forming a weak encounter complex in a disordered conformation and is then subsequently locked-in by a folding step. (Reprinted with permission from [128]); Copyright 2014 American Chemical Society (**D**) Model of the partly disordered MeV N_TAIL_–P_XD_ complex as a conformational ensemble as derived from a combined EPR and modeling approach. 50 best-fit structures of the 488–525 region of N_TAIL_ in complex with P_XD_. The N_TAIL_ conformers are depicted with a color gradient ranging from yellow to red with increasing structural density, while P_XD_ is shown in black (modified from [129]). A typical EPR spectrum is also shown; and (**E**) Cartoon representation of the structural model of the N_TAIL_–P_XD_ complex as derived from a combined ESI-IM-MS and modeling approach. The disordered N_TAIL_ is shown in blue and the ordered X domain of P is shown in orange. A typical MS spectrum is shown. Reprinted with permission from [122]. Copyright 2014 The American Society for Mass Spectrometry. EPR: electron paramagnetic resonance, MS: mass spectrometry, MoRE: Molecular Recognition Element, MD: molecular dynamics. Structures were drawn using Pymol [124].

Similarly to HeV and NiV, MeV N_TAIL_ undergoes α-helical folding upon binding to P_XD_. The P_XD_-induced α-helical folding occurs within the predicted α-MoRE located within Box2 (amino acids 486–504) and gives rise to a pseudo-four helix complex of which the crystal structure has been solved at 1.8 Å resolution (Figure 5A) [35,36]. In the structure of the chimeric construct in which P_XD_ and the α-MoRE are covalently linked to each other, the α-MoRE of N_TAIL_ adopts a parallel orientation with respect to P_XD_ and is embedded in a large hydrophobic cleft delimited by helices α2 and α3 [36]. Indeed, the residues that are involved in the interaction are mainly hydrophobic, involving Leu481, Leu484, Ile488, Phe497, Met500, and Ile504 from P_XD_ and Ser491, Ala494, Leu495, Leu498 and Met501 from N_TAIL_.

The dynamics of the MeV N_TAIL_–P_XD_ complex in solution has been thoroughly investigated in SDSL EPR, SAXS and NMR studies [85,86,87,130]. Indeed, in SDSL EPR studies, 14 single-site MeV N_TAIL_ cysteine variants were designed, purified and labeled and their EPR spectra were recorded in the presence or absence of P_XD_ [85,86]. The mobility of the spin labels grafted within the 488–502 (*i.e.*, Box2) and 505–522 regions was found to be severely and moderately reduced, respectively, upon addition of P_XD_ [86]. The restrained motion of the 505–522 region upon binding to P_XD_ was shown to be due to the α-helical transition occurring within the neighboring Box2 region and not to a direct interaction with P_XD_ nor to gain of regular secondary structure [86]. ITC studies further supported the lack of contribution of Box3 in the interactions with P_XD_ by showing that the removal of Box3 does not affect the binding strength of the N_TAIL_–P_XD_ interaction [80].

The parallel orientation of the MoRE at the P_XD_ surface is thus a conserved feature between MeV and HeV, arguing for a functional relevance. It is tempting to speculate that this could be related to the relative orientation of the whole P protein with respect to the N_TAIL_ region protruding from the nucleocapsid. In its turn, this might be related to optimal positioning of the polymerase onto the nucleocapsid template and might impart directionality to the polymerase movement along the nucleocapsid.

Another common feature among MeV, HeV and, supposedly, NiV N_TAIL_–P_XD_ complexes is the hydrophobic nature of their interface, in line with the findings by Meszaros and co-workers who reported that the binding interfaces of protein complexes involving IDPs are often enriched in hydrophobic residues [131]. Burying of hydrophobic residues of the MeV α-MoRE at the P_XD_ surface is thought to provide the driving force of its induced folding. We therefore modeled the more hydrophobic side of the amphipathic α-MoRE of *Henipavirus* N_TAIL_ at the hydrophobic surface delimited by helices α2 and α3 of P_XD_ using the MeV N_TAIL_–P_XD_ structure as a template. The two modeled complexes display a rather small interface area in agreement with previous reports indicating that the interfaces of complexes involving IDPs are generally smaller than those occurring in ordered complexes [70]. Strikingly, in the case of the SeV N_TAIL_–P_XD_ complex, charged residues dominate the interface, thus giving a good illustration of how selection pressure allowed the C-terminal domains of N and P to evolve concomitantly within the *Paramyxoviridae* family leading to protein complexes having the same 3D fold and the same function, but with very limited sequence identity [34].

From a mechanistic point of view, two extreme mechanisms of IDPs-based interactions have been described in the literature (see [29] and references therein cited). According to these two mechanisms, IDP/Rs can fold either before or upon binding to the partner. In the first case, the partner binds to an already pre-folded MoRE through a conformational selection mechanism, thereby shifting the conformational ensemble to the folded form [132,133]. In the second mechanism, folding occurs upon binding, where the interaction with the partner induces the gain of structure within the MoRE, very often through hydrophobic contacts. However, a mixed mechanism involving both induced folding (*i.e.*, folding after binding) and conformational selection (*i.e.*, folding before binding) has been often reported [134].

In spite of the subtle differences distinguishing the *Henipavirus* and MeV N_TAIL_–P_XD_ complexes, they share similar structural features, thus suggesting a possible common folding coupled to binding mechanism. Although the occurrence of a transiently populated α-helix even in the absence of the partner would suggest that the molecular mechanism governing the P_XD_-induced folding of N_TAIL_ could rely on conformer selection (*i.e.*, selection by the partner of a pre-existing conformation), quantitative analysis of NMR titration data obtained for the MeV N_TAIL_–P_XD_ binding reaction [87] indicate that the binding reaction may also involve a binding intermediate in the form of a weak, non specific encounter complex hence implying also an induced folding mechanism [135]. A combined mechanism of conformational selection and induced folding was further supported by data obtained by MD simulations. Indeed, a synergistic mechanism in which the recognition event proceeds via (minor) conformational selection, followed by (major) induced folding has been proposed (Figure 5B) [84]. Interestingly, recent kinetic experiments on the MeV N_TAIL_–P_XD_ binding reaction allowed the identification of at least two kinetic steps and revealed unambiguously that the reaction occurs via a folding after binding scenario, whereby the dominant pathway occurs via the accumulation of a partially folded intermediate followed by a subsequent monomolecular folding event (Figure 5C) [128].

Similarly to MeV N_TAIL_, NMR and SDSL EPR data [63,64,83] support the presence within *Henipavirus* N_TAIL_ domains of a pre-formed α-MoRE in the free state. The extent to which this pre-configuration may presage the bound form and/or favor conformational selection is expectedly different between NiV and HeV given that the NiV α-MoRE is more populated and long-lived than its HeV counterpart [63,64,83]. In the case of HeV, quantitative analysis of peak intensities in the HSQC spectra of N_TAIL_ at each P_XD_ titration point showed that the signal intensity decreases faster for the residues located at the extremities of the α-MoRE and for which a smaller amount of residual helical structure is observed in the isolated state of N_TAIL_. This differential broadening suggests that P_XD_ binds to a short, central helix within the α-MoRE, and that this helix is subsequently extended via helical folding of the adjacent residues. Data therefore indicate that N_TAIL_ interacts with P_XD_ via a folding-upon-binding mechanism, with the folding event occurring on the micro- to millisecond time scale [63]. Definite conclusions as to whether the higher helical sampling by the NiV N_TAIL_ α-MoRE imparts a different mechanism of folding coupled to binding to P_XD_ await direct experimental data that are so far missing.

From a functional point of view, the N_TAIL_–P_XD_ interaction is thought to trigger the opening of the nucleocapsid to provide access of the polymerase to the viral RNA, which is tightly protected by the assembled N. In agreement, EM studies showed that addition of P_XD_ triggers unwinding of MeV nucleocapsids (Bhella and Longhi, unpublished data). This dramatic conformational change is accompanied by an increased exposure of viral RNA to the solvent as indicated by its increased sensitivity to RNAse. In line with these observations, recent studies documented the ability of the MuV P protein to induce nucleocapsid uncoiling, with both N- and C-terminal P domains being involved [136]. In striking contrast with these findings, NMR studies have shown that addition of P_XD_ to HeV nucleocapsids does not trigger any major nucleocapsid rearrangement, as judged from the fact that the only peaks that are affected by the addition of the partner are those of the residues belonging to the α-MoRE [63]. These findings provide evidence that the environment of N_TAIL_ is identical in the free and the P_XD_-bound form within the nucleocapsids, thereby supporting the absence of major unwinding or rearrangements of the nucleocapsids [63]. The expectedly necessary nucleocapsid unwinding might require either the full-length P protein, or the P–L complex and/or cellular cofactors. One such a possible cellular cofactor could be hsp70, one of the experimentally confirmed MeV N_TAIL_ partners [92,93,94]. The hypothesized ability of hsp70 to promote nucleocapsid conformational changes is corroborated by previous studies on the closely related canine distemper virus, where hsp70-nucleocapsid complexes were found to exhibit an expanded helical diameter, an increased fragility, and an enhanced exposure of the genomic RNA to nuclease degradation [137,138].

A tight N-P complex is predicted to hinder polymerase processivity according to the cartwheeling model. This model posits that contacts between N_TAIL_ and P_XD_ have to be dynamically made/broken to allow the polymerase to progress along the nucleocapsid template in order to allow transcription and replication to take place. In support of a relationship between affinity of the N_TAIL_–P_XD_ and polymerase processivity, recent data obtained using a quantitative mammalian protein complementation assay and recombinant measles viruses allowing the conditional expression of wild-type or mutated *P* genes, showed that an increase in the MeV N_TAIL_–P_XD_ binding strength resulting from P_XD_ substitutions is associated with a slower transcript accumulation rate and that abolishing the interaction renders the polymerase non functional [139]. In striking contrast with these data, previous mutational studies that targeted the Box2 region of MeV N_TAIL_ showed that a reduced binding strength has no impact on the polymerase rate [140]. Collectively, the results provided by these two independent studies suggest that while increasing the N_TAIL_–P_XD_ binding strength has an effect on the polymerase rate, decreasing it (without abrogating it) has no impact. We can speculate that this tolerance of the polymerase to N_TAIL_ substitutions leading to a reduced binding strength is probably true only in a certain range of affinities, where in spite of a pronounced drop in the affinity towards P_XD_, the N_TAIL_–P_XD_ interaction remains strong enough to ensure recruitment of the polymerase. In all case, the results provided by the mutational study that targeted Box2 clearly point to the need of revisiting the accepted model whereby the N_TAIL_–P_XD_ interaction has to be relatively weak to allow the polymerase to cartwheel on the nucleocapsid template. Indeed, a relatively labile complex can result either from an inherently lower affinity of the binding reaction, or from a tight complex whose strength is modulated by co-factors. Hsp70 is one such an experimentally confirmed modulator in the case of MeV. In fact, hsp70 binds to the same MeV N_TAIL_ sites as P_XD_ and thus competes out this latter [93]. It is, therefore, tempting to speculate that the progression of the MeV polymerase complex along the template could be ensured by hsp70. In this model hsp70 would promote successive cycles of binding and release thanks to its destabilizing effect on the N_TAIL_–P_XD_ interaction.

### 4.2. Fuzziness of N_TAIL_–P_XD_ Complexes

“Fuzziness” is a paradigm of protein structure and function that emerged from the observation that the ordering of an IDP upon binding to a target is often not complete, *i.e.*, a significant residual structural disorder can persist in the complex [28]. In other words, an IDP could either sample a number of conformations at the surface of the partner (*i.e.*, static fuzziness) or preserves polypeptide extensions in the disordered state (*i.e.*, dynamic fuzziness). In this latter case, the flexible chains are usually not involved in complex formation but could serve for instance as tails for partner fishing with non-specific, transient contacts [141,142]. The abundance of such complexes in the literature supports the functional significance of residual structural disorder in macromolecular complexes.

The N_TAIL_–P_XD_ complexes from paramyxoviruses provide illustrative examples of a combination of both static and dynamic fuzziness. Indeed, the induced α-MoRE within *Henipavirus* N_TAIL_ domains was found to remain highly dynamic at the surface of P_XD_, *i.e.*, to sample many sub-conformations reflecting a static fuzzy complex. On the other hand, the remainder of the N_TAIL_ chain remains flexible within the three complexes thus supporting the formation of a dynamic fuzzy complex. These findings have been confirmed through several lines of evidences as described below (see also [143]).

For the three N_TAIL_ domains, the majority of the peaks display chemical shifts that are nearly unaltered upon addition of the partner [63,64,87,90,91] indicating that the majority of N_TAIL_ remains disordered in the bound form. In line with that, the experimentally determined R_S_ of the NiV N_TAIL_–P_XD_ complex (35.4 Å) suggests that binding to P_XD_ does not imply formation of a compact complex (expected R_S_ = 22.3 Å), with the resulting complex rather retaining a considerable flexibility [90]. In further support of the “fuzziness” within *Henipavirus* and MeV N_TAIL_–P_XD_ complexes, EPR results pointed out lack of involvement of MeV Box1 and of *Henipavirus* Box1 and Box4 in complex formation, with the labels grafted within these Boxes retaining a high mobility and solvent accessibility [83,86]. In the same vein, intrinsic fluorescence spectroscopy data showed lack of P_XD_ impact on the environment of a Trp residue introduced within Box4, thus further supporting a high conformational freedom and solvent exposure of the C-terminal region of *Henipavirus* N_TAIL_ [90]. The NMR behavior of *Henipavirus* N_TAIL_, where some resonances disappear upon addition of P_XD_ and never come back even at saturation [63,64,90], suggests that even when bound to P_XD_ N_TAIL_ remains dynamic, undergoing exchange between different conformers at the P_XD_ surface [63]. SAXS studies of MeV N_TAIL_–P_XD_ complex provided a low-resolution model of the N_TAIL_ bound form, which showed that most of N_TAIL_ (amino acids 401–488) remains disordered within the complex [91]. Furthermore, by combining SDSL EPR spectroscopy and modeling, an ensemble description of the structure of the MeV N_TAIL_ region encompassing amino acids 489–525 bound to P_XD_ could be obtained, which revealed that the region downstream the MoRE remains highly flexible (Figure 5D) [129]. Interestingly, a recent study combining ESI-IM-MS and modeling provided structural models of the MeV N_TAIL_–P_XD_ complexes at the atomistic level (Figure 5E). These complexes are characterized by different levels of compaction, thus further supporting their structural heterogeneity [122]. Beyond documenting structural heterogeneity, those studies enabled to capture a collapsed form of the complex that had escaped detection in previous studies. Indeed, a bimodal charge state distribution was observed with a high-charge component (18+) and a low-charge (11+) component. While the former would correspond to an “open” conformation, in which the disordered arms of N_TAIL_ flanking the α-MoRE fluctuate maintaining high solvent accessibility, the low-charge component likely represents a compact or “closed” conformation of the complex in which the N_TAIL_ arms collapse onto the surface of the folded partner [122]. Computational modeling of the “open” complex in solution, using experimental chemical shifts as restraints, provided atomic-resolution structural models with calculated solvent accessible surface area (SASA) in good agreement with that experimentally determined by ESI-MS. In the resulting models, the intermolecular interactions are predominantly hydrophobic, not only in the ordered core of the complex, but also in the disordered regions.

Many functional advantages can result from fuzziness, including interactions with alternative partners and simultaneous interactions with different partners. Indeed, the residual plasticity often allows adaptation of the same motif to different partners, or a variable arrangement of the recognition motifs, which can mediate interactions with alternative partners (*i.e.*, promiscuity). Moreover, the disordered tails in complexes can serve for partner fishing via non-specific, transient contacts. Fuzzy parts of the complexes can harbor regulatory PTM sites. They can even directly or indirectly interfere with (promote or inhibit) binding of the part that undergoes folding transition. In addition, fuzziness provides a way to reduce the entropic penalty that accompanies the disorder-to-order transition, thereby affording enhanced affinity. Tuning fuzziness therefore provides an additional way to modulate the interaction strength.

In line with these expectations, the fuzzy Box3 region of MeV N_TAIL_ was shown to serve as a binding site for hsp70 [93,94], where the latter is known to stimulate both viral transcription and replication [92,144,145]. Box3 constitutes however a low-affinity binding site for hsp70, with Box2 providing a high-affinity binding site (*K*_D_ of 10 nM) [92,146]. Since hsp70 competitively inhibits P_XD_ binding to N_TAIL_ [93], hsp70 could enhance transcription and genome replication by reducing the stability of N_TAIL_–P_XD_ complexes [91,93]. The hsp70-dependent reduction of the stability of P-N_TAIL_ complexes would thus rely on competition between hsp70 and P_XD_ for binding to the α-MoRE of N_TAIL_, with recruitment of hsp70 being ensured by both Box2 and Box3 [93].

As already mentioned, PTM is over-represented in IDPs [45,147], and disorder-to-order transitions occur in some cases after PTMs, such as phosphorylation, thus supporting a significant role of PTMs in regulating the functions of IDPs [45]. Interestingly, in the case of MeV, phosphorylation of the N protein has been shown to upregulate the transcriptional activity of minigenomic RNA and to regulate viral genomic RNA stability [148,149]. Likewise, in the case of NiV, phosphorylation of N was found to be involved in the regulation of viral RNA synthesis, with rapid turnover of phosphorylation being critical [150]. Two residues located within MeV N_TAIL_, namely S479 and S510, have been found to constitute the major phosphorylation sites within MeV N, while in the case of NiV only one phosphorylated residue, namely S451, was identified [150]. Interestingly, these residues are all located in fuzzy regions of N_TAIL_, which is in good agreement with the three structural requirements of PTMs: an appropriate local sequence, structural exposure, and flexibility of the site so that it can be productively accommodated by the active site of the modifying enzyme [45,147].

Finally, fuzzy regions flanking MoREs can also serve as natural modulators of the interactions established by IDPs. In fact, a recent random mutagenesis study of MeV N_TAIL_ led to the identification of five regulatory regions that are located in the N-terminal fuzzy region of N_TAIL_ and dampen the interaction [151]. This finding is consistent with recent observations based on mini-replicon studies that unveiled that the region upstream Box2 acts as a negative modulator for the binding of the polymerase complex [104]. In the same vein, MeV N_TAIL_ variants devoid of Box3 were found to exhibit enhanced interaction with P_XD_, suggesting that Box3 would naturally serve as a dampener (see Section 4.3).

### 4.3. Molecular Determinants of the Affinity of the N_TAIL_–P_XD_ Interaction as Unveiled by Random Mutagenesis

In the last decade, a wealth of bioinformatics and experimental studies showed that intrinsic disorder enhances protein interactivity, with IDPs and IDRs being able to bind several partners while maintaining both specificity and binding efficiencies. However, the molecular features of the binding efficiency of IDP/Rs (*i.e.*, un/coupling between affinity and specificity) is far from being elucidated (see [152] for a review on this topic). In order to shed light onto these aspects, we applied a combinatorial experimental approach on the MeV N_TAIL_–P_XD_ complex, which not only provided further perspicacity on the N_TAIL_–P_XD_ complex, but also proved to be a valuable general approach to characterize complexes involving IDPs/IDRs.

This approach, termed “descriptive random mutagenesis”, is an unbiased method that relies on targeting the sequence of an IDP/IDR, in this case MeV N_TAIL_, by random mutagenesis thus allowing to assess how amino acid substitutions introduced at random affect partner recognition. Subsequent to the generation of a library of N_TAIL_ random mutants, the interaction strength towards P_XD_ was evaluated using a protein complementation assay based on green fluorescent protein (GFP) reassembly [153].

In this assay, each partner is fused to a GFP moiety and the interaction between the two proteins drives the re-assembly of the two GFP fragments thus producing a fluorescence signal that is directly related to the binding strength [154]. Besides confirming previous results on the crucial role of the α-MoRE in binding to P_XD_, the obtained results provided novel insights by dissecting the N_TAIL_ region and identifying regulatory segments within the fuzzy parts of N_TAIL_ [154].

In that study, 224 variants out of 300, which were randomly chosen without any selection pressure, were found to encode full-length forms of N_TAIL_ with the substitutions providing a full coverage of the whole length of the N_TAIL_ sequence (*i.e.*, each amino acid of the sequence was found to be substituted at least once). Interestingly, most of these substitutions were shown to affect the N_TAIL_ capacity to bind P_XD_. Substitutions within Box2 (*i.e.*, the P_XD_-binding site) were found to lead to a reduced fluorescence, thus indicating that Box2 is poorly evolvable in terms of binding abilities towards P_XD_. The critical positions leading to the highest decrease in the fluorescence were found to correspond to residues with side chains oriented to the partner. The study led however also to the identification of Arg497 as an additional critical Box2 residue for stabilizing the N_TAIL_–P_XD,_ whose role in complex formation had escaped detection in previous studies. Although the side chain of Arg497 points out of the binding surface, it is located at bonding distance from the hydroxyl group of Tyr480 of P_XD_. Through generation and characterization of a “mirror” P_XD_ variant bearing the Y480F substitution, the crucial role of the Arg497-Tyr480 interaction in stabilizing the N_TAIL_–P_XD_ complex was confirmed [154].

Beyond Box2, the study also allowed the identification of five regulatory regions, termed e-boxes (enhancer-boxes), located in the fuzzy region upstream Box2. Mutating these regions leads to enhanced interaction, indicating that e-boxes act as natural dampeners of the interaction. The precise mechanism by which they exert this role remains however unknown.

Random mutagenesis of N_TAIL_ also resulted in the generation of truncated variants (*i.e.*, 76 out of 300 variants) arising from the insertion of a stop codon. In line with expectations, variants devoid of Box2 showed a dramatic drop in the fluorescence, reflecting loss of interaction. Most interestingly, variants that are only devoid of Box3 were found to display an increased fluorescence, thus unveiling an inhibitory effect of Box3 on the interaction with P_XD_.

In conclusion, this study unveiled that most of the N_TAIL_ sequence is sensitive to mutations and possesses a few regulatory sites located within fuzzy regions. The fuzziness of N_TAIL_ may therefore not only serve as a way to capture other binding partners but also to modulate the strength of interactions established by N_TAIL_.

## 5. N-P Interactions as Promising Targets for Anti-Paramyxoviral Approaches

In the last years, it has become increasingly evident that inhibition of protein–protein interactions (PPIs) is endowed with a great therapeutic potential. While most drugs available on the market target the active site of enzymes [155] or ligand binding sites of receptors [156], targeting PPIs is very attractive since protein interaction surfaces are much less conserved offering the potential for highly specific inhibition. Consequently, the inhibition of PPIs has emerged during the last decade, from both academic and private research, as a new way to modulate the activity of proteins. The first set of drug-like compounds that functionally target the HIV-1 Nef-SH3 binding surface has recently provided the “proof of concept” for antiviral discoveries relying on PPI inhibition, thus paving the way towards a new class of antiviral molecules [157].

Strategies to prevent viral infection could target any of the three steps of viral multiplication (*i.e.*, attachment/fusion, replication/transcription and assembly/budding). Although undoubtedly viral entry is a valuable target for antiviral strategies that benefited from the identification of virus host cell receptors and from the elucidation of the molecular mechanisms leading to membrane fusion, a promising way to inhibit paramyxovirus replication consists in targeting the N–P interaction due to its crucial role in both transcription and replication. As discussed above, in *Paramyxovirinae*, recruitment of L onto the nucleocapsid template relies on the N_TAIL_–P_XD_ interaction, whereas encapsidation of nascent genome requires the dual N_TAIL_–P_XD_ and N°–P_NTD_ interaction within the N°–P complex.

That the N°–PNT interaction is a valuable target of antiviral approaches is well illustrated by the antiviral activity of a peptide targeting the interaction between the unassembled form of the nucleoprotein (N°) and P_NTD_ in both NiV [66] and RSV [158]. The relevance of the N_TAIL_–P_XD_ and N°–PNT interactions, both involving a structured partner and an IDR, as possible targets of antiviral drugs is further underscored by recent reports, showing that PPIs involving IDRs are valuable drug discovery targets with the potential to increase significantly the discovery rate for new compounds [159,160,161,162].

Disruption of interactions between IDPs and globular proteins seems particularly feasible because of their different mode of interaction. Indeed PPIs involving one structured partner and one disordered partner have many features that make them potentially druggable. IDPs often bind their partner through MoREs that bind grooves or clefts of the partner. In this way, the interface resembles the type of interaction observed in receptor-ligand or enzyme-substrate binding, which can often be targeted by small molecules. Interfaces of IDPs are slightly smaller than those of ordered complexes: their average surface is of 1141 ± 110 Å^2^ [70] while for globular proteins the surface is larger with an average area of 1600 ± 400 Å^2^ [163]. Another typical feature lies in the number of structural regions involved in the interaction. In the case of globular proteins, distinct segments are brought together to participate in the creation of binding sites, with the primary sequence regions contacting a partner protein being often discontinuous [131]. In contrast, IDP interfaces often only involve a single sequentially continuous segment. Finally, a further difference is that while globular proteins contribute most of their hydrophobic residues to the protein core, IDPs expose their few hydrophobic residues to the surface to allow interaction with binding partners. 40%–90% of IDP hydrophobic residues are exposed to the surface *vs.* only 5%–15% for ordered proteins [131]. Therefore IDP interfaces rely more on hydrophobic contacts (33% for IDPs and 22% for ordered proteins) whereas ordered proteins make more polar-polar interactions [131,164]. This latter feature makes IDP interfaces more druggable by small hydrophobic compounds, which represents a potential advantage in terms of their expected cell permeability. That IDP interfaces are druggable is supported by the identification of several small molecule drugs targeting PPIs between disordered and ordered partners (see [161,162] and references therein cited). For example, promising small compounds have been found to bind to the groove of Mdm2, thereby blocking its interaction with a disordered region of p53 [165,166,167].

The N_TAIL_–P_XD_ and N°–P_NTD_ interactions are attractive targets as, beyond involving a disordered partner, they are endowed with a number of features that support their potential druggability. The rather weak binding affinity of the N_TAIL_–P_XD_ interaction (*K*_D_ in the μM range) is expected to allow tighter competitive binding by small molecule drugs to the structured partner. As for the N°–P_NTD_ interaction, although the K_D_ has not been determined, this interaction is physiologically dynamic, being competitively inhibited by the viral RNA for encapsidation of the nascent RNA chain. As such, the complex is not expected to be very tight.

The N_TAIL_–P_XD_ interface was shown to be relatively small (*i.e.*, <700 Å^2^) in the case of MeV [35]. It is predicted to be even smaller (*i.e.*, <450 Å^2^) in the case of *Henipavirus* complexes [90]. The small interface area presages an interaction that is prone to destabilization, in agreement with the commonly accepted relationship between interface buried surface area and complex stability [168]. Comparatively, the total interface area of the NiV N°–P_NTD_ complex is much larger (1440 Å^2^), in line with the larger size of the P region embedded at the N surface. It should be noted however, that the P_NTD_ region does not constitute a continuous structural segment, being rather composed of two helices separated by a kink [66]. Each of the two binding interfaces can be targeted individually.

In spite of the additional role of electrostatics in complex formation, as a matter of fact, the N_TAIL_–P_XD_ interface in MeV and in Henipaviruses relies on hydrophobic contacts and a recent survey of protein–protein interfaces with known inhibitors pointed out that these interfaces are more hydrophobic than general PPI’s interfaces, with less charged residues and more non-polar atoms [169]. In addition, binding of N_TAIL_ to P_XD_, and of the N-terminal α-MoRE of PNT to N° involves embedding of an α-helix in a hydrophobic cleft of the structured partner, which should facilitate mimicry with small molecules.

Last, but not least, since *Henipavirus* N_TAIL_ domains are functionally interchangeable with respect to their ability to bind P_XD_ [80], a single inhibitor could probably target both interactions thus paving the way towards a new set of broad-range antivirals.

## 6. Conclusions

When we analyzed the modular organization of the P proteins within the *Paramyxovirinae* subfamily, we noticed that a larger PNT domain in *Henipavirus* P proteins accounts for the extra length of their P protein with respect to other paramyxoviruses [30]. This finding is consistent with the higher tolerance of disordered regions to insertions or major rearrangements as compared to ordered ones. Moreover, since the P-encoded proteins are believed to possess anti-interferon functions, the extension in *Henipavirus* P proteins might have evolved to better equip these viruses so as to enhance their capacity to overcome the cellular interferon response. Furthermore, the disordered nature of PNT and of the “spacer” region connecting PNT to PMD likely reflects a way of alleviating evolutionary constraints within overlapping reading frames. Indeed, PNT partially overlaps with the C protein (being encoded by the same RNA region), and the “spacer” region partially overlaps with the C-terminal domain of the V protein [30,31]. This observation is in agreement with previous reports pointing out a relationship between overlapping genes and structural disorder [31,170,171,172,173]. We thus reasoned that structural disorder, which is encoded by a much wider portion of sequence space as compared to order, can indeed represent a strategy by which genes encoding overlapping reading frames can lessen evolutionary constraints imposed on their sequence by the overlap, allowing the encoded overlapping protein products to sample a wider sequence space without losing function.

Following our seminal studies that pointed out the abundance of disorder in *Paramyxovirinae* N and P proteins [31,32,57,174], several subsequent studies have documented the prevalence of disorder in viral proteins using both computational and experimental approaches (see [175] and references therein cited). Bioinformatics studies showed that viral proteins, and in particular proteins from RNA viruses, have a high disorder content [176,177]. In those studies, the authors propose that beyond affording a broad partnership, the wide occurrence of disordered regions in viral proteins could also be related to the typical high mutation rates of RNA viruses, *i.e.*, it could represent a strategy for buffering the deleterious effects of mutations.

A detailed comparative examination of viral and non-viral proteins showed that, amongst several distinguishing characteristics, viral proteins possess (i) a larger fraction of residues that are not organized into regular secondary structural elements; (ii) conformational stabilities that are less affected by mutations; (iii) a high rate of mutations; (iv) enrichment in proteins encoded by overlapping reading frames; and (v) a higher content of polar residues. These features indicate that they have been shaped by evolution to be endowed with better adaptation to their hostile habitats and to rapid changes in their biological and physical environment. Indeed, one of the many noteworthy features of viruses is their ability to adapt to very harsh and hostile environments and to adjust themselves according to the biological and genetic features of the hosts, which in turn are often adapted to exist at extreme conditions (see [175] and references therein cited). The above-mentioned features are interestingly in intimate relationship with structural disorder, which indeed provides several advantages.

In fact, because viruses are obligate intracellular parasites, they have to interact with various components of the host, including membranes, nucleic acids, and proteins. The lack of a rigid 3D structure imparts to IDP/Rs the necessary plasticity to establish various interactions with several partners at once. In the course of evolution, viruses have “learned” to hijack and manipulate host proteins for their benefit, and to evade the host defense mechanisms. A recent study by Davey and co-workers showed that viruses have achieved this ability through broad mimicry of host protein short linear motifs (SLiMs) [178], where the latter are embedded in disordered regions and play a variety of roles, including targeting host proteins for proteosomal degradation, cell signaling, directing proteins to the correct subcellular localization, deregulating cell cycle checkpoints, and altering transcription of host proteins [179]. Importantly, binding to cell proteins through sites that mimic SLiMs also helps viral proteins in eluding the host cell’s immune system, by rendering viral epitopes poorly recognizable by the host immune system (see [175] and references therein cited).

Based on all these considerations, we proposed that the main advantage of the abundance of disorder within viral proteins would reside in pleiotropy and genetic compaction [175]. Indeed, disorder provides a solution to reduce both genome size and molecular crowding, where a single gene would (i) encode a single (regulatory) protein product that can establish multiple interactions via its disordered regions and hence exert multiple concomitant biological effects including evasion of the host immune response; and/or (ii) would encode more than one product by means of overlapping reading frames.
